# Arg-GlcNAcylation on TRADD by NleB and SseK1 Is Crucial for Bacterial Pathogenesis

**DOI:** 10.3389/fcell.2020.00641

**Published:** 2020-07-17

**Authors:** Juan Xue, Shufan Hu, Yuxuan Huang, Qi Zhang, Xueying Yi, Xing Pan, Shan Li

**Affiliations:** ^1^Taihe Hospital, Institute of Infection and Immunity, Hubei University of Medicine, Shiyan, China; ^2^College of Life Science and Technology, Huazhong Agricultural University, Wuhan, China; ^3^College of Biomedicine and Health, Huazhong Agricultural University, Wuhan, China

**Keywords:** enteropathogenic *Escherichia coli*, T3SS effectors, NleB, SseK, arginine GlcNAc transferase, TRADD

## Abstract

Death receptor signaling is critical for cell death, inflammation, and immune homeostasis. Hijacking death receptors and their corresponding adaptors through type III secretion system (T3SS) effectors has been evolved to be a bacterial evasion strategy. NleB from enteropathogenic *Escherichia coli* (EPEC) and SseK1/2/3 from *Salmonella enterica* serovar Typhimurium (*S.* Typhimurium) can modify some death domain (DD) proteins through arginine-GlcNAcylation. Here, we performed a substrate screen on 12 host DD proteins with conserved arginine during EPEC and *Salmonella* infection. NleB from EPEC hijacked death receptor signaling through tumor necrosis factor receptor 1 (TNFR1)-associated death domain protein (TRADD), FAS-associated death domain protein (FADD), and receptor-interacting serine/threonine-protein kinase 1 (RIPK1), whereas SseK1 and SseK3 disturbed TNF signaling through the modification of TRADD Arg235/Arg245 and TNFR1 Arg376, respectively. Furthermore, mouse infection studies showed that SseK1 but not SseK3 rescued the bacterial colonization deficiency contributed by the deletion of NleBc (*Citrobacter* NleB), indicating that TRADD was the *in vivo* substrate. The result provides an insight into the mechanism by which attaching and effacing (A/E) pathogen manipulate TRADD-mediated signaling and evade host immune defense through T3SS effectors.

## Introduction

Death receptor signaling is crucial for cell death (Giogha et al., 2014; Luo et al., 2015; Galluzzi et al., 2018), inflammation (Park et al., 2007), and immune homeostasis (Wilson et al., 2009). It is mediated by homotypic or heterotypic interactions among death domains (DDs) of the TNFR family of transmembrane death receptors and the downstream adaptors, including TRADD (Hsu et al., 1995; Luo et al., 2015), RIPK1 (Stanger et al., 1995; Luo et al., 2015), and FADD (Chinnaiyan et al., 1995; Luo et al., 2015). TRADD is known as the initial adaptor for TNFR1-induced apoptosis and nuclear factor-κB (NF-κB) signaling (Hsu et al., 1995; Chen and Goeddel, 2002; Mak and Yeh, 2002; Chen et al., 2008; Pobezinskaya et al., 2008; Pobezinskaya and Liu, 2012). Recent studies have found that TRADD has a Goldilocks effect on the survival of *Ripk1^–/–^Ripk3^–/–^* mice. Both *Tradd^+/+^Ripk1^–/–^Ripk3^–/–^*and *Tradd^–/–^Ripk1^–/–^Ripk3^–/–^* mice result in death through apoptosis, while a single allele of TRADD is optimal for survival of *Ripk1^–/–^Ripk3^–/–^* mice (Dowling et al., 2019). RIPK1 and TRADD are synergistically required for TRAIL-induced NF-κB signaling and TNFR1-induced NF-κB signaling and apoptosis (Fullsack et al., 2019). Besides that, TRADD plays roles independent of TNFR1 signaling, such as downstream of Toll-like receptors (Chen et al., 2008; Pobezinskaya et al., 2008) and DR3 (Chinnaiyan et al., 1996; Kitson et al., 1996; Pobezinskaya et al., 2011; Pobezinskaya and Liu, 2012). Type III secretion system effector NleB from enteropathogenic *E. coli* (EPEC) was previously reported as an arginine GlcNAc transferase that inhibited multiple death receptor mediated inflammation and cell death by modifying a conserved arginine residue in some death domain proteins (Li et al., 2013; Pearson et al., 2013; Ding et al., 2019; Pan et al., 2020; Xue et al., 2020). The arginine GlcNAc transferase activity of NleB is critical for attaching and effacing (A/E) pathogen colonization in the mouse colon (Li et al., 2013; Pearson et al., 2013; Scott et al., 2017; Ding et al., 2019). Although modification of TRADD, FADD, and RIPK1 in *in vitro* reconstitution system and *ex vivo* epithelial cell infection system has been studied, the *in vivo* substrate preference of NleB remains elusive.

Intracellular pathogen *Salmonella entrica* strains secreted three *Salmonella* pathogenicity island 2 (SPI-2) effector SseK1, SseK2, and SseK3 (Kujat Choy et al., 2004; Brown et al., 2011; Baison-Olmo et al., 2015; El Qaidi et al., 2017; Gunster et al., 2017; Yang et al., 2018; Araujo-Garrido et al., 2020; Meng et al., 2020). Crystal structure studies show that NleB, SseK1, and SseK3 belong to the GT-A family glycosyltransferase (Esposito et al., 2018; Park et al., 2018; Ding et al., 2019; Araujo-Garrido et al., 2020; Pan et al., 2020). The crystal structures of NleB in complex with FADD-DD and the sugar donor, and NleB-GlcNAcylated DDs (TRADD-DD and RIPK1-DD) show that NleB is an inverting enzyme. NleB converts the α-configuration in the UDP-GlcNAc donor into the β-configuration toward the conserved arginine of DD proteins, namely, TRADD Arg235, FADD Arg117, and RIPK1 Arg603 (Ding et al., 2019; Xue et al., 2020). Previous *in vitro* studies have suggested that SseK1 could GlcNAcylate TRADD (Li et al., 2013; Gunster et al., 2017; Xue et al., 2020), FADD (Gunster et al., 2017), and GAPDH (Gao et al., 2013; El Qaidi et al., 2017) with different efficiency. However, the substrate specificity of SseK effectors remains controversial.

Therefore, this study applied a substrate screen of 12 conserved arginine-containing DD proteins during EPEC and *Salmonella* infection, finding that SseK1 and SseK3 selectively modify TRADD and TNFR1, respectively. SseK1 GlcNAcylated hTRADD at Arg235 and Arg245 while SseK3 targeted TNFR1 at Arg376. SseK1 but not SseK3 can inhibit TRADD-activated NF-κB and apoptosis. Taking advantage of the substrate specificity of SseK effectors, we found that only chimera SseK1 fully rescued the bacterial colonization deficiency contributed by the deletion of NleBc in *Citrobacter rodentium* (*C. rodentium*) infection animal model. This result indicates that TRADD is the preferred *in vivo* substrate corresponding to NleB/SseK1-induced bacterial virulence. More importantly, the TRADD^–/–^ mice infection model confirmed this result. All these findings suggest that arginine GlcNAcylation in TRADD catalyzed by type III-translocated bacterial effector proteins NleB and SseK1 is crucial for the pathogenesis of A/E pathogen.

## Materials and Methods

### Bacterial Strains and Growth Conditions

The EPEC strains, *Salmonella* strains, and *C. rodentium* strains used in this study, unless specially mentioned, were grown in LB broth at 37°C, shaking with the following antibiotics: nalidixic acid (50 μg/ml) (0677, AMRESCO), kanamycin (50 μg/ml) (1758-9316, INALCO), ampicillin (100 μg/ml) (1758-9314, INALCO), chloramphenicol (17 μg/ml) (1758-9321, INALCO), and streptomycin (50 μg/ml) (1758-9319, INALCO).

### Plasmid Construction

*nleB* gene and *sseK1/2/3* genes were amplified from EPEC E2348/69, *C. rodentium* ICC168, and *S. enterica* Typhimurium SL1344 strains. These genes were inserted into pCS2-EGFP, pCS2-1Flag, and pCS2-3Flag for mammalian cell expression, and into pGEX-6P-2 and pET28a-His for protein expression in *E. coli*. The pTRC99A vector was used for complementation in EPEC (under the trc promoter) and the pET28a vector for complementation in *C. rodentium* (under the *C. rodentium nleB* signal peptide) and *S.* Typhimurium (with their upstream promoter regions). Human cDNAs for TRADD, TNFR1, FADD, DR3 DD, ANK1 DD, ANKDD1B, ANK2 DD, ANK3 DD, RIPKI DD, FAS DD, DR4 DD, DR5 DD were amplified from a HeLa cDNA library as previously described (Li et al., 2013). All single point mutants were generated by quick change and multiple point mutants and truncation mutants were generated by standard molecular biology procedures. NF-κB reporter plasmids were used as previously described (Li et al., 2007, 2013). All plasmids were verified by DNA sequencing and primers were synthesized by Sangon Biotech.

### Antibodies and Reagents

The anti-GlcNAc arginine antibody (ab195033, Abcam) was described previously (Pan et al., 2014). Antibody for Flag M2 (F2426) and tubulin (T5186) were Sigma products. Antibodies for EGFP (sc8334) and DnaK 8E2/2 (ab69617) were purchased from Santa Cruz Biotechnology and Abcam, respectively. Horse radish peroxidase (HRP)-conjugated goat anti-mouse IgG (NA931V) and HRP-conjugated goat anti-rabbit IgG (NA934) were both from GE Healthcare. Unless specially mentioned, the cell culture products were purchased from Invitrogen, and all other reagents were Sigma-Aldrich products.

### Recombinant Protein Expression and Purification

Protein expression was induced overnight in *E. coli* BL21 (DE3) strain at 22°C with 0.4 mM isopropyl-β-D-thiogalactopyranoside (IPTG) when OD600 reached 0.8~1.0. Affinity purification of GST-TRADD DD and the site-directed mutants expression alone or co-expression with SseK1 were performed by using glutathione sepharose (GE Healthcare, United States), following the manufactures’ instructions. Proteins were further purified by ion exchange chromatography. All the purified recombinant proteins were concentrated and stored in the buffer containing 20 mM Tris-HCl (pH 8.0), 150 mM NaCl, and 5 mM dithiothreitol. The protein purity was examined by SDS-PAGE, followed by Coomassie Blue staining.

### Cell Culture and NF-κB Luciferase Reporter Assay

293T cells and HeLa cells obtained from the American Type Culture Collection (ATCC) and MEF cells provided by S. Ghosh were grown in DMEM (GIBCO) medium supplemented with 10% FBS (GIBCO and BI), 2 mM L-glutamine (GIBCO), 100 U/ml penicillin, and 100 mg/ml streptomycin (GIBCO). These cells were cultivated at 37°C in the presence of 5% CO_2_. Vigofect (Vigorus) was used for 293T cell transfection and jetPRIME (PolyPlus) for HeLa cell transfection, following the respective manufacturer’s instructions. Luciferase activity was determined 24 h after transfection by using the dual luciferase assay kit (Promega) according to the manufacturer’s instructions.

### Immunoprecipitation

The 293T cells seeded in 6-well plates at a confluency of 60–80% were transfected with a total of 5 μg plasmids. Twenty-four hours after transfection, cells were washed in phosphate-buffered saline (PBS) and lysed in lysis buffer A containing 25 mM Tris-HCl, pH 7.6, 150 mM NaCl, 10% glycerol, and 1% Triton, supplemented with a protease inhibitor cocktail (Roche). Cells were collected and centrifuged under 13,200 rpm at 4°C for 15 min. Pre-cleared lysates were subjected to anti-Flag M2 immunoprecipitation following the manufacturer’s instructions. After a 2 h incubation, the beads were washed four times with lysis buffer B containing 25 mM Tris-HCl, pH 7.6, 150 mM NaCl, 10% glycerol, and 0.5% Triton, and the immunoprecipitates were eluted by 1 × SDS sample buffer, followed by standard immunoblotting analysis. All the immunoprecipitation assays were performed more than three times, and representative results are shown in figures.

### Bacterial Infection of Mammalian Cells and Cell Death Assay

Bacterial infection and cell death were performed as described previously (Li et al., 2013; Gunster et al., 2017; Ding et al., 2019; Newson et al., 2019; Xue et al., 2020). Briefly, MEF cells were seeded in 96-well plates at a concentration of 2 × 10^4^ per well one day before infection. A single colony in 0.5 ml LB was incubated overnight in a static LB culture at 37°C. Bacterial strains were then diluted by 1:40 in antibiotic-free DMEM supplemented with 1 mM IPTG and cultured at 37°C in the presence of 5% CO_2_ for an additional 4 h to OD600 approximately at 0.4∼0.6. Infection was performed at a multiplicity of infection (MOI) of 200 in the presence of 1 mM IPTG for 2 h, with centrifugation at 800 g for 10 min at room temperature to promote infection. After the infection, cells were washed four times with PBS, and the extra bacteria were killed with 200 μg/ml gentamicin. One-hour CHX pretreatment (2 μg/ml) was used to sensitize TNF (20 ng/ml)-stimulated cell death. Cell survival was then determined 15 h after treatment with TNF by using the CellTiter-Glo Luminescent Cell Viability Assay kit (Promega).

### Liquid Chromatography-Mass Spectrometry Analysis of Intact Proteins

The method was performed as described previously (Xue et al., 2020). In brief, 10 μg of purified protein was injected and separated by reversed-phase liquid chromatography in a Dionex Ultimate 3000 HPLC system (Thermo Fisher Scientific, United States). Using a C4 capillary column (MAbPacTM RP, 4 μm, 2.1 × 50 mm, Thermo Fisher Scientific, United States), the flow rate was set at 0.3 ml/min and the linear 10 min gradient of buffer A (0.1% Formic acid) and buffer B (0.1% Formic acid, 80% acetonitrile) was from 5 to 100%. The eluted proteins were sprayed into a Q Exactive Plus mass spectrometer (Thermo Fisher Scientific, United States) equipped with a Heated Electrospray Ionization (HESI-II) Probe (Thermo Fisher Scientific, United States). Thermo Scientific Protein Deconvolution program was used to analyze the mass accuracy of the intact protein.

### Mice Infection and *C. rodentium* Colonization Assays

All animal experiments were conducted following the Chinese National Ministry of Health guidelines for housing and care of laboratory animals, and the experiments were performed in accordance with institutional regulations made by the Institutional Animal Care and Use Committee at Taihe Hospital, Hubei University of Medicine. The 5–6 weeks old of C57BL/6 male mice and TRADD^–/–^ mice were maintained in the specific pathogen-free environment. All the mice were randomly divided into each experimental group with no blind mice and were housed individually in high-efficiency particulate air (HEPA)-filtered cages with sterile bedding. Independent experiments were performed using 6–8 mice per group. The mice infection was performed as described previously (Li et al., 2013; Ding et al., 2019).

### Caspase-3 Activity Assay

The activity of prepared cell lysates toward Ac-DEVD-AFC were conducted as previously described (Meng et al., 2016; Wang et al., 2020). Briefly, 293T cells transfected with the indicated plasmid combinations were washed and lysed. The pre-cleared cell lysates collected were mixed with Na-Citrate buffer (50 mM Tris-HCl, pH 7.4, 1 M Na-Citrate, 10 mM DTT, 0.05% CHAPS). Peptide substrates Ac-DEVD-AFC for caspase-3 was added to each well to a final concentration of 20 μM to start the reaction. Assay plates were incubated at 37°C and the caspase-3 activity was measured every 2 min for total 2 h. Substrate cleavage was monitored by measuring the emission signal at 510 nm wavelength by a 405 nm fluorescence excitation on a microplate reader (SpectraMax i3x, Molecular Devices). Fluorometric assays were conducted in white opaque tissue culture plates and all measurements were carried out in triplicate.

### Statistical Analysis

All the values of at least three independent experiments were presented. Statistical analysis was performed using Student’s *t*-test. The comparison of multiple groups was conducted by using one-way analysis of variance (ANOVA) or two-way ANOVA. *P* < 0.05 was considered significant.

## Results

### SseK1 and SseK3 Selectively Modify TRADD and TNFR1 During Pathogen Infection

**FIGURE 1:**
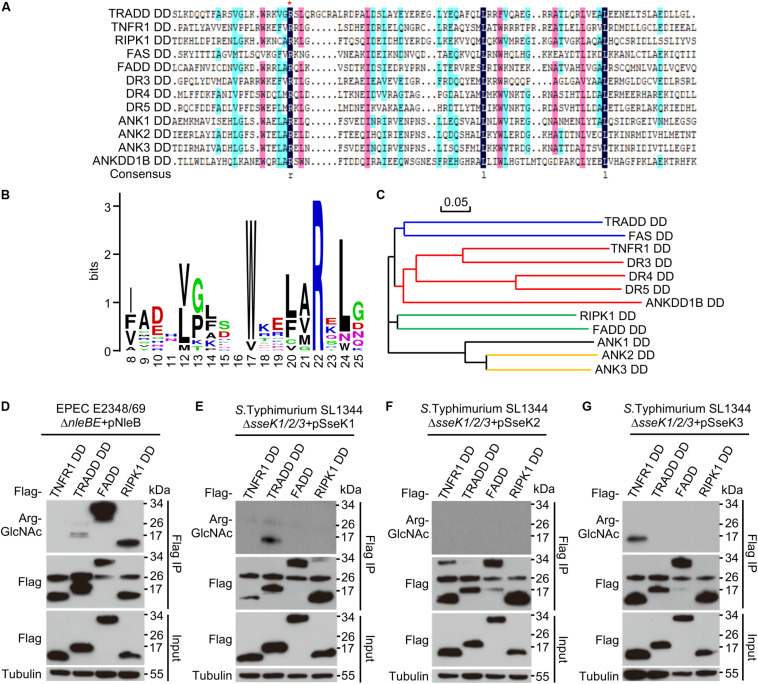
SseK1 and SseK3 selectively modify TRADD and TNFR1 during pathogen infection. **(A)** Multiple sequence alignment of 12 DD proteins from 34 human DD-containing proteins. The red asterisk indicates the conserved arginine. **(B)** Site distribution of amino acids in 12 conserved arginine containing-DDs. **(C)** Phylogenetic tree of the 12 DDs constructed on the basis of sequence similarity. **(D–G)** Selective GlcNAcylation of DDs by NleB and SseK1/2/3 during bacterial infection. 293T cells transfected with the indicated Flag-DD plasmids were infected with EPEC or *Salmonella* strains. Cell lysates were subjected to anti-Flag immunoprecipitation and immunoblotting. Data in **(D–G)** are from at least three independent experiments.

Death domain (DD) is a subclass of protein motif known as the death fold (Park et al., 2007). DD-containing proteins are usually associated with programmed cell death and inflammation (Park et al., 2007; Park, 2011; Ferrao and Wu, 2012). Multiple sequence alignment of DDs in human genome revealed that Arg235 in TRADD (Arg117 in FADD) was conserved in one-third of the total 37 DD-containing proteins, including TNFR1, TRADD, FADD, RIPK1, FAS, DR3, DR4, DR5, ANK1, ANK2, ANK3, and ANKDD1B (Figures 1A,B and Supplementary Figure 1). The phylogenetic tree of the 12 DDs showed an evolutionary relationship based on protein sequence (Figure 1C). Previous studies show that the conserved arginine is critical for NleB mediated modification (Li et al., 2013; Ding et al., 2019; Pan et al., 2020). Here, we screened all the arginine-containing DD proteins to be GlcNAcylated by NleB/SseKs in bacterial pathogen infection systems. Arginine GlcNAc transferase-deficient strains from EPEC and *Salmonella* were generated. NleB was expressed in EPEC strains, while SseK1, SseK2, and SseK3 were individually expressed in *Salmonella* strains. NleB from EPEC E2348/69 modified TRADD DD, FADD, and RIPK1 DD (Figure 1D and Supplementary Figure 2). SseK1 and SseK3 from *S. Typhimurium* SL1344 specifically modified TRADD DD and TNFR1 DD, respectively, while SseK2 exhibited no obvious arginine GlcNAcylation activity toward DD proteins during infection (Figures 1E–G and Supplementary Figure 2). Other arginine containing death domains could not be modified (Supplementary Figure 2).

### SseK1 GlcNAcylates TRADD at Arg235 and Arg245

**FIGURE 2:**
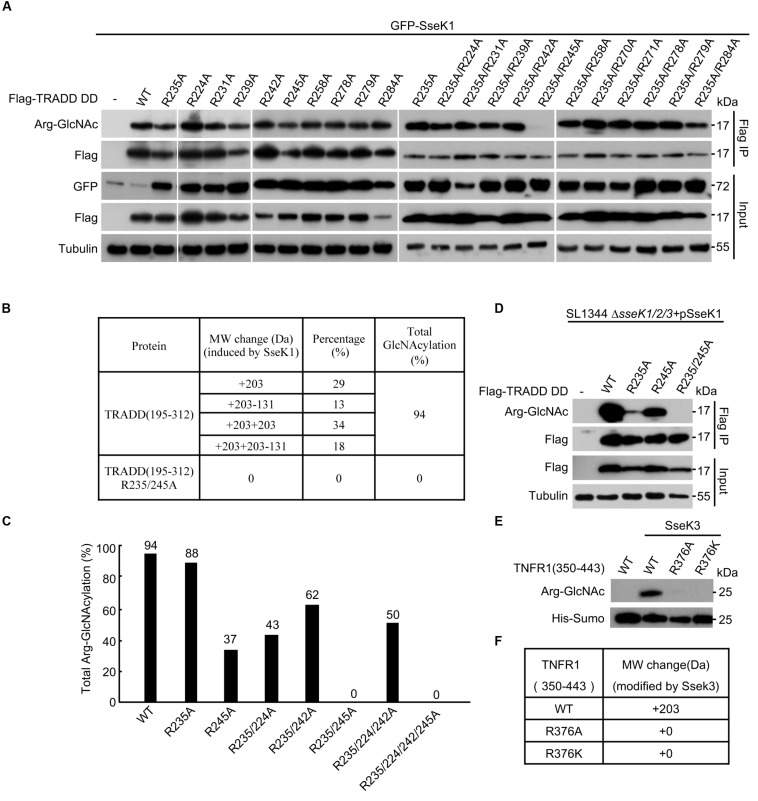
SseK1 GlcNAcylates hTRADD at R235/R245and SseK3 GlcNAcylates TNFR1 at R376. **(A)** An arginine point mutation screen of hTRADD to investigate its ability to be GlcNAcylated by SseK1. 293T cells were transfected with the indicated plasmid combinations. The samples of anti-Flag immunoprecipitates (Flag IP) and total cell lysates (Input) were immunoblotted with the corresponding antibodies. Anti-tubulin was used as a loading control. **(B)** Mass spectrometry summary of the total molecular weight of TRADD DD and TRADD DD (Arg235Ala/Arg245Ala) mutant co-expressed with SseK1 in bacteria. “ + 203” indicates a single GlcNAc modification. “ + 203-131” indicates in addition to a single GlcNAc modification, one methionine is missing as well. “ + 203 + 203” indicates two GlcNAc modifications. “ + 203 + 203-131” indicates in addition to two GlcNAc modifications, one methionine is missing as well. **(C)** Total Arg-GlcNAcylation modification percentages of TRADD and TRADD arginine mutants catalyzed by SseK1. **(D)** Modification of TRADD and TRADD variants by SseK1 upon *S.* Typhimurium infection. Plasmids carrying genes for Flag-TRADD DD, Flag-TRADD DD R235A, Flag-TRADD DD R245A, and Flag-TRADD DD R235/245A transfected into 293T cells were infected with the indicated *Salmonella* strains. After infection, cells were lysed and proteins were immunoprecipitated with Flag beads, followed by standard immunoblotting analysis with the indicated antibodies. **(E)** TNFR1 WT and Arg376 mutants co-expressed with SseK3 in BL21. Bacterial lysates were subjected to SDS-PAGE, followed by immunoblotting analyses as shown. **(F)** Summary of ESI-MS determination of the total mass of TNFR1 and its mutants co-expressed with SseK3 in bacteria. Data in **(A–D)** are from at least three independent experiments.

Arg235 was the only modification site on TRADD by NleB (Li et al., 2013). However, mutation of Arg235 in TRADD could not abolish the modification of human TRADD by SseK1 (Figure 2A). This was in accordance with the previous report on mouse TRADD (Gunster et al., 2017), suggesting that SseK1-mediated GlcNAcylation of TRADD occurred on either a different residue or on multiple arginine residues. We applied an arginine point mutation screen of human TRADD to explore the modification sites of TRADD by SseK1. 293T cells were co-transfected with SseK1 and wild-type or different point mutation variants of TRADD. Single point mutation of any arginines in the death domain of TRADD had no effects on the modification detected by the Arg-GlcNAc antibody (Figure 2A). Subsequently, we mutated all the arginine residues on the background of Arg235Ala mutation and found that GlcNAcylation of TRADD Arg235Ala/Arg245Ala mutant was significantly reduced in western blot (Pan et al., 2014; Figure 2A). To determine the modification ratio, recombinant TRADD DD and its mutants were co-expressed with SseK1 in *E. coli* BL21 (DE3) strain. Purified proteins were analyzed on the mass spectrometer, and the summary of the resulting total molecular weight of mass spectra was shown (Figures 2B,C). Electrospray ionization mass spectrometry (ESI-MS) analysis identified one GlcNAc moiety addition and two GlcNAc moieties addition on TRADD DD, suggesting two sites can be modified simultaneously. Double mutation of Arg235 and Arg245 exhibited the theoretical molecular weight (Figure 2B). Consistently, SseK1 delivered from a *Salmonella* derivative strain SL1344Δ*sseK1/2/3* by T3SS, could not modify TRADD DD (R235A/R245A) yet, even though single arginine mutation could be modified (Figure 2D). For the modification of TNFR1 by SseK3, mutation of Arg376 into Ala or Lys abolished the arginine-GlcNAcylation signal and molecular weight increase, suggesting that the conserved Arg376 was the bona fide modification site (Figures 2E,F). All these data showed that SseK1 GlcNAcylated hTRADD at Arg235 and Arg245 both in the co-expression system and in the pathogen infection process.

### SseK1 Inhibits TRADD DD-Activated NF-κB and Cell Death Signaling in 293T Cells

**FIGURE 3:**
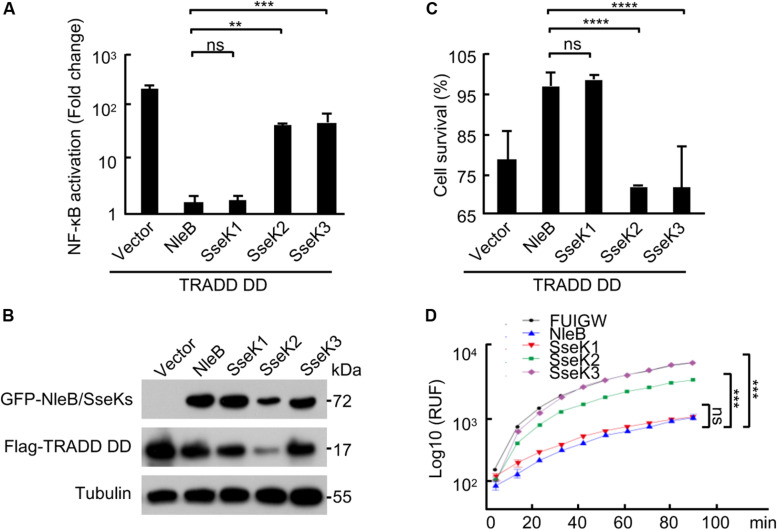
SseK1 inhibits TRADD-activated NF-κB and cell death signaling. **(A–D)** Effects of NleB/SseK on TRADD DD-activated NF-κB **(A,B)**, cell survival **(C)**, and caspase-3 activation **(D)**. 293T cells were transfected with Flag-TRADD DD in combination with the indicated plasmids. NF-κB luciferase activation was indicated as fold change **(A)**. Cell viability was determined by measuring ATP levels **(C)**. Caspase-3 activity was assayed using an Ac-DEVD-AFC probe according to manufacturer’s instructions **(D)**. For A and C, one-way ANOVA was used for statistical analysis. And for D, two-way ANOVA was used. **P < 0.01, ***P < 0.001, ****P < 0.0001, ns, not statistically significant. All data are acquired at the same time and at least three independent experiments.

Overexpression of TRADD protein can activate both NF-κB and cell death signaling (Li et al., 2013). SseK1 significantly abolished TRADD DD overexpression-induced NF-κB activation (Figures 3A,B), cell death (Figure 3C), and caspase-3 activation in 293T cells (Figure 3D). However, SseK3 had no effects on these TRADD DD-activated signaling pathways (Figures 3A–D). Thus, SseK1 could target TRADD directly and disrupt multiple signaling pathways at the downstream of TRADD.

### Chimera SseK1 and SseK3 Inhibits TNF-Induced Cell Death During *C. rodentium* Infection

**FIGURE 4:**
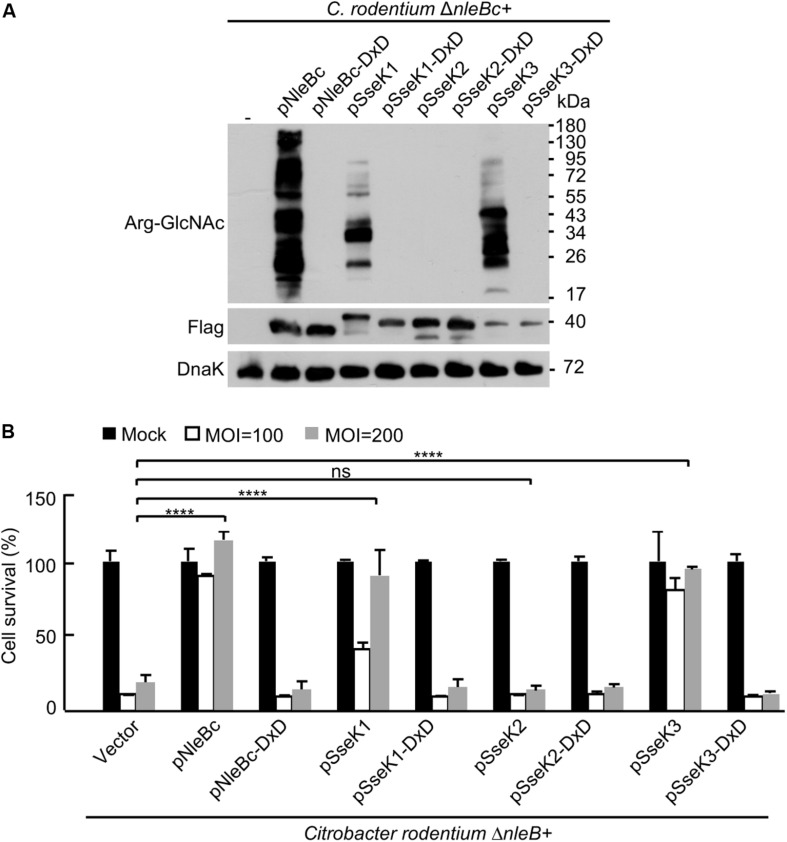
Chimera SseK1 and chimera SseK3 inhibits TNF-induced cell death during *C. rodentium* infection on MEF cells. **(A)** Complementation strains of Δ*nleB* with *Citrobacter* NleBc or chimera SseK1/2/3 and their enzyme inactivated mutants were generated. Single colony of each strain was inoculated and incubated overnight with the corresponding antibiotics. Bacterial lysates were collected and samples were loaded onto SDS-PAGE gels, followed by standard immunoblotting analysis. **(B)** NleB and SseK1/3 mediate inhibition of TNF-induced cell death in MEF cells during *C. rodentium* infection. MEF cells infected with derivatives of *C. rodentium* strains were stimulated with TNF. Cell viability was determined by measuring ATP levels. ****P < 0.0001 (one-way ANOVA), ns, not statistically significant. Data are representative from at least three repetitions.

Considering the broad substrate range of NleB during cell culture infection, it is intriguing to determine the target in *in vivo* infection system. Signal peptides of SseK1/2/3 were replaced with a signal peptide of NleBc to make the chimera SseK1/2/3, which harbor the substrate specificity and can be secreted as NleBc. Complementation strains of Δ*nleB* with *Citrobacter* NleBc or chimera SseK1/2/3 were generated. Chimera SseK1/2/3 expressed normally in *C. rodentium* strain. Chimera SseK1 and SseK3 exhibited their enzymatic activity to modify the substrates in bacteria, whereas the DxD mutants did not (Figure 4A). During MEF cell infection, complementation with chimera SseK1 and SseK3, but not SseK2, or respected DxD mutants, inhibited TNF-induced cell death. This inhibition was similar to the level of complementation of NleB (Figure 4B), which indicated chimera SseK1 and SseK3 were both translocated and played enzymatic roles in MEF cells. Collectively, these results suggested that chimera SseK1 and SseK3 were functionally secreted during *C. rodentium* infection.

### Chimera SseK1 but Not Chimera SseK3 Rescues the Function of NleB in Animal Infection Model

**FIGURE 5:**
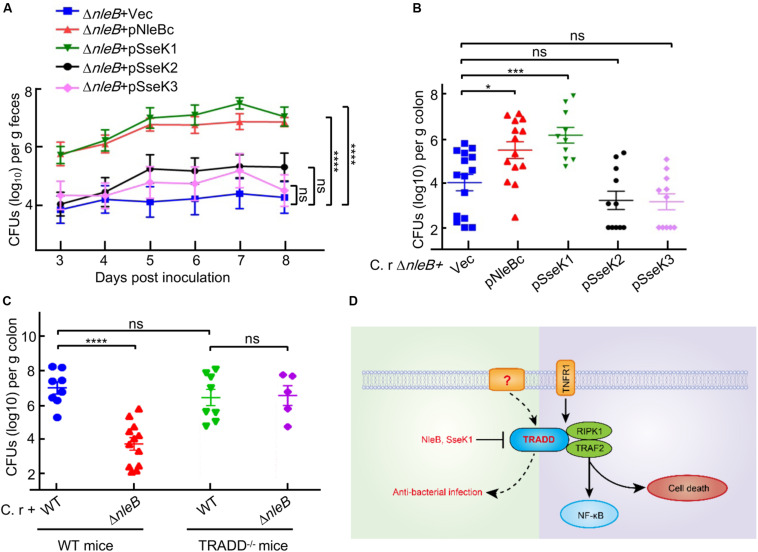
Only SseK1 fully rescues the bacterial colonization contributed by NleBc in *C. rodentium* infection animal model. **(A,B)** 5–6 week-old C57BL/6 male mice were orally gavaged with the indicated *C. rodentium* derivatives. The viable stool bacteria count (log10 CFU/g feces) **(A)** and bacterial colonization in the colon (log10 CFU/g colon, *n* > 6) 8 days post-infection **(B)** were presented as mean ± s.e.m. pNleB(c), pSseK1, pSseK2, and pSseK3 are complementary plasmids expressing NleB(c), SseK1, SseK2, and SseK3, respectively. **(C)** 5–6 week-old TRADD^–/–^ mice and parent mice were orally gavaged with wild type *C. rodentium* and *nleB* deletion mutant. Bacterial colonization in the colon (log10 CFU/g colon, *n* > 6) 8 days post-infection were presented. *P < 0.05, ***P < 0.001, and ****P < 0.0001 (one-way ANOVA), ns, not statistically significant. Data in **(A–C)** are representative from at least three repetitions. **(D)** Schematic diagram of TRADD-mediated signaling.

Infection of mice with *C. rodentium* is a natural and physiologically relevant model to study pathogen-host interactions for A/E pathogens (Kamada et al., 2012; Li et al., 2013; Pearson et al., 2013; Collins et al., 2014; Crepin et al., 2016; Ding et al., 2019). Previous studies showed that the arginine GlcNAc transferase activity of NleB was crucial for bacterial colonization and virulence in mice infection model (Li et al., 2013; Pearson et al., 2013; Ding et al., 2019). We performed mouse infection assays to investigate the preferred *in vivo* substrate of NleB by utilizing the substrate specificity of SseK1/3. In *C. rodentium*-inoculated C57BL/6 mice, the Δ*nleB* mutant showed a significantly reduced colonization compared to the wild-type strain, demonstrated by colony-forming units of bacteria recovered from stool samples and colons from infected mice. Complementation of Δ*nleB* with NleBc or SseK1, but not SseK2 or SseK3, recovered stool counts to the level of the wild-type strain and restored bacterial colonization in the intestinal tract of mice (Figures 5A,B). SseK1 exhibited substrate specificity to TRADD, indicating TRADD was the preferential *in vivo* substrate corresponding to NleB-induced bacterial virulence. We hypothesized that if NleB interfered with TRADD-mediated signaling during infection, then *C. rodentium* Δ*nleB* mutant bacteria would no longer be attenuated in mice with defects in TRADD. Indeed, TRADD^–/–^ mice infected with the Δ*nleB* mutant showed comparable levels of bacterial colonization compared to wild-type mice infected with wild-type *C. rodentium* (Figure 5C).

## Discussion

Previous studies confirmed that the arginine GlcNAc transferase activity of NleB was essential for bacterial colonization in the mouse model of EPEC infection (Li et al., 2013; Pearson et al., 2013; Ding et al., 2019). Considering that NleB could modify the DDs of TRADD, FADD, and RIPK1 in cell culture infection system (Li et al., 2013; Lu et al., 2015; Scott et al., 2017; Ding et al., 2019; Xue et al., 2020), it is necessary to determine the target *in vivo*. In *C. rodentium* infection animal model, we found that only SseK1 fully rescued the bacterial colonization deficiency contributed by NleB from *Citrobacter* or EPEC. The finding that SseK1 had substrate specificity to TRADD indicated that TRADD was the preferential *in vivo* substrate corresponding to NleB-induced bacterial colonization. TRADD^–/–^ mice infection model confirmed this result, which might be correlated with its role of dynamic equilibrium in both cell death and NF-κB signaling. SseK3 disrupted TNF signaling by directly targeting TNFR1, which was the upstream receptor of TRADD. Inhibition of TRADD but not TNFR1 was beneficial for bacterial colonization, thus this provided insights into the non-TNFR1 signaling role of TRADD (Figure 5D; Chen et al., 2008; Pobezinskaya et al., 2008).

In summary, this study presented compelling evidence for distinct substrate specificities of NleB, SseK1, SseK2, and SseK3 among 12 death domains. SseK1 fully rescued the bacterial colonization deficiency contributed by NleBc in *C. rodentium* infection animal model. TRADD knockout diminished the colonization attenuation effect of NleB deletion. All the data suggest that TRADD is the preferential *in vivo* substrate corresponding to NleB-induced bacterial colonization.

## Author’s Note

This manuscript has been released as a Pre-Print at bioRxiv (Xue et al., 2019).

## Data Availability Statement

All datasets presented in this study are included in the article/Supplementary Material.

## Ethics Statement

The animal study was reviewed and approved by the Ethics Committee of Huazhong Agricultural University.

## Author Contributions

SL and JX conceived the overall study, designed the experiments, and wrote the manuscript. JX, SH, and YH conducted and performed the majority of the experiments, analyzed data with assistance from QZ, XP, and XY. All authors read and approved the final version of the manuscript.

## Conflict of Interest

The authors declare that the research was conducted in the absence of any commercial or financial relationships that could be construed as a potential conflict of interest.
