# Supplementary material for: Arg-GlcNAcylation on TRADD by NleB and SseK1 Is Crucial for Bacterial Pathogenesis
Source: Front Cell Dev Biol. 2020 Jul 17;8:641. doi: 10.3389/fcell.2020.00641 (PMC7379376; doi:10.3389/fcell.2020.00641)
Supplement: Supplementary file 1 [file Table_1.DOCX]

Supplementary Material


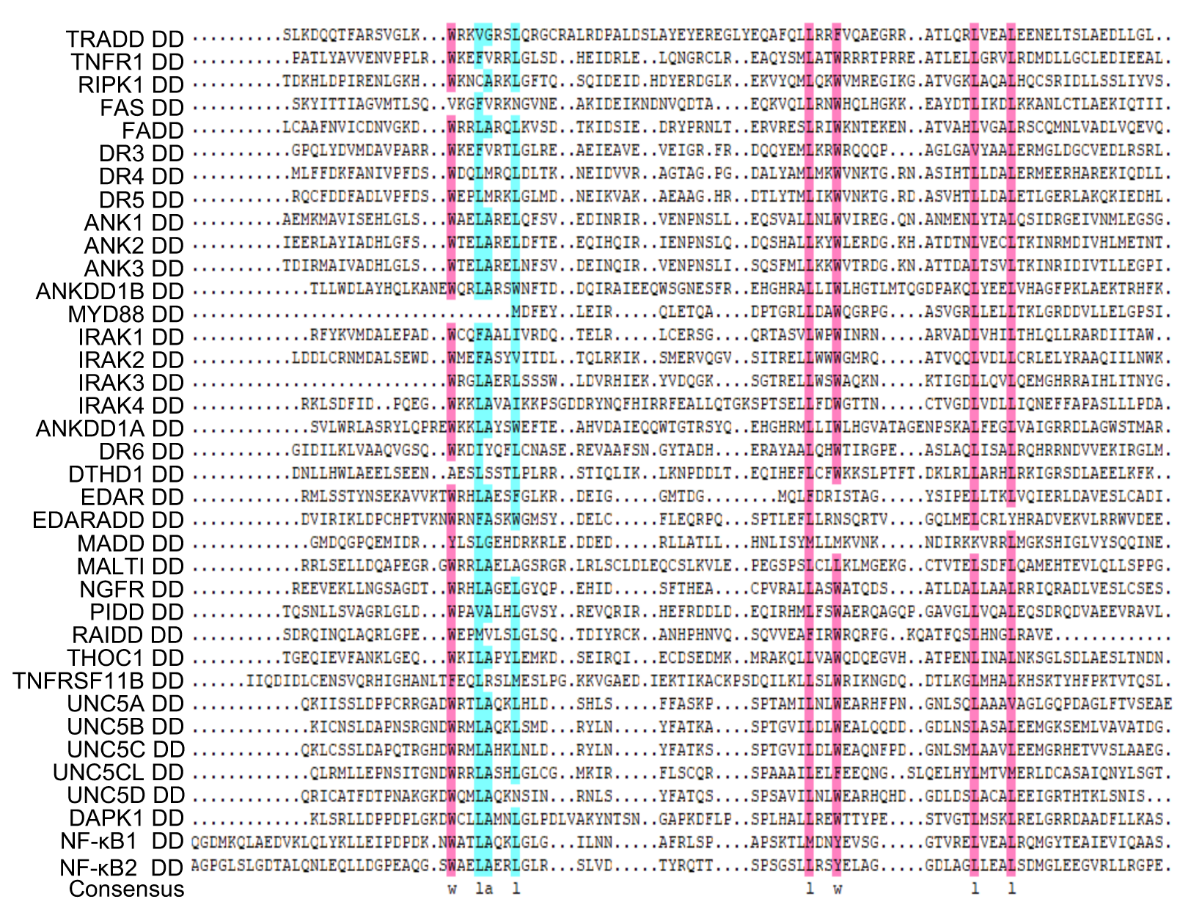


**Supplementary Figure 1 Multiple sequence alignment of 37 DDs from human death domain proteins.** The alignment was performed using the software DNAMAN8.


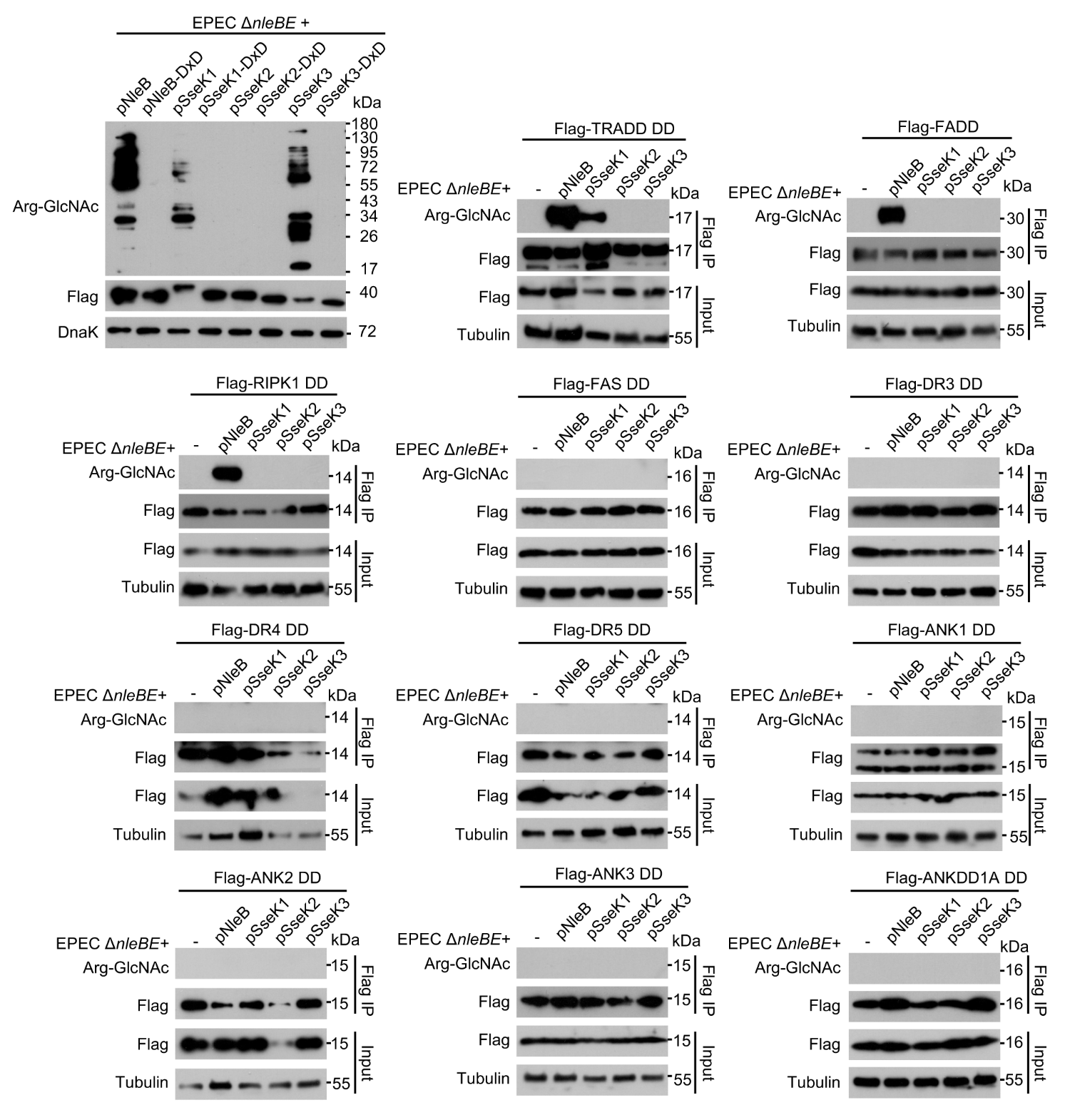


**Supplementary Figure 2 Arginine-GlcNAcylation profile of 12 human DDs by NleB and chimera SseKs during EPEC infection.** 293T cells expressing Flag-DDs were infected with indicated EPEC mutants that were complemented with plasmids expressing wild type NleB or chimera SseK1/2/3. After infection, cells were lysed, and proteins were immunoprecipitated with anti-Flag beads. Samples were loaded onto SDS-PAGE gels and were immunoblotted with anti-Flag, anti-Arg-GlcNAc, and anti-tubulin. Blot data are derived from at least three independent experiments.
